# The relationship of serum 25-hydroxyvitamin D with glucose homeostasis in obese children and adolescents in Zhejiang, China

**DOI:** 10.1186/1687-9856-2015-S1-O49

**Published:** 2015-04-28

**Authors:** Ke Huang, You-Jun Jiang, Jun-Fen Fu, Jian-Feng Liang, Hong Zhu, Li-Fei Hu, Zhi-Wei Zhu, Guan-Pin Dong, Xue-Feng Chen

**Affiliations:** 1 Department of Endocrinology, Children’s Hospital of Zhejiang University School of Medicine, Hangzhou, Zhejiang, China; 2 Department of Statistics, Children’s Hospital of Zhejiang University School of Medicine, Hangzhou, Zhejiang, China; 3 Department of Child Health Care, Children’s Hospital of Zhejiang University School of Medicine, Hangzhou, Zhejiang, China

## Aims

Evidence of the association between vitamin D, insulin resistance and oral disposition index (oDI) in obese children and adolescents is limited. We investigated serum 25(OH) D levels in obese children and adolescents in Zhejiang, China, and determined the relationship between serum 25(OH) D and glucose metabolism.

## Method

A cross-sectional design was used. All together 348 obese and 445 non-obese children and adolescents (aged from 6-16 years old) were enrolled in this study. Obese children were divided into four subgroups: normal glucose tolerance (NGT), isolated impaired fasting glucose (IFG), isolated impaired glucose tolerance (IGT), combined IFG and ITG (IFG+ITG) according to the oral glucose tolerance test. We measured serum 25(OH) D levels and calculated the homeostasis model of insulin resistance (HOMA-IR), the whole body insulin sensitivity index (WBISI), the product of β-cell function and insulin sensitivity by the disposition index (DI).

## Results

The levels of 25(OH)D in obese group were significantly lower than those of non-obese group; serum 25(OH)D level in obese with NGT group was higher than that of the other three subgroups, and it was significantly inversed with LogHOMA-IR (r=-0.114, p=0.035), positively correlated with LogWBISI, LogHOMA0DI after control for age, sex, season, puberty stage (r=0.111, p=0.040; r=0.122, p=0.024). Obese patients with vitamin D deficiency have a significantly higher risk of disturbing the glucose metabolism, such as IFG, ITG, IFG plus ITG, either IFG or ITG, for its OR 3.198(95%CI 1.467-6.97), 5.443(95%CI 1.863-15.897), 5.560(95%CI 1.212-25.502), 4.007(95%CI 2.017-7.962).

## Conclusion

25(OH) D deficiencies or insufficiency are common in obese children and adolescents in Zhejiang, China. Obese patients with 25(OH) D deficiency (<30nmol /L) are at higher risk for abnormal glucose metabolism.

